# Ciliated muconodular papillary tumor with a growing cavity shadow that mimicked colorectal metastasis to the lung: a case report

**DOI:** 10.1186/s40792-020-01012-7

**Published:** 2020-09-29

**Authors:** Kotaro Murakami, Yojiro Yutaka, Naoki Nakajima, Akihiko Yoshizawa, Hiroshi Date

**Affiliations:** 1 Department of Thoracic Surgery, Kyoto University Hospital, 54 KawaharachoSakyo-ku, ShogoinKyoto, 606-8507 Japan; 2 Diagnostic Pathology, Department of Thoracic Surgery, Kyoto University Hospital, Kyoto, Japan

**Keywords:** Ciliated muconodular papillary tumor, Cavity shadow, Metastatic lung tumor, Non-palpable tumor

## Abstract

**Background:**

Ciliated muconodular papillary tumor (CMPT) is a rare papillary nodule tumor with benign and malignant characteristics that occurs in the peripheral lung.

**Case presentation:**

A 70-year-old woman who underwent right hemicolectomy for colorectal cancer (CRC; pT3N0M0, p-stage II) 2 years prior, presented with a sub-centimeter growing cavity shadow on chest computed tomography (CT), which was suspected to be a CRC metastasis. Because positron emission tomography CT suggested there was no other site suspicious of recurrence, thoracoscopic resection with preoperative pleural dye marking was planned to remove the small lesion, which seemed to be hardly palpable on CT. Immediately after pleural dye marking adjacent to the lesion using cone beam CT in the hybrid operating room, thoracoscopic wedge resection was performed and the tumor was finally diagnosed as CMPT, characterized by the papillary growth of mucus-producing cells in the alveoli.

**Conclusion:**

We resected the non-palpable small lung lesions following preoperative marking using cone-beam CT in the hybrid operating room. This case highlights a rare cavitary CT image of a CMPT mimicking a metastatic lung tumor from colorectal cancer.

## Background

Ciliated muconodular papillary tumor (CMPT) is a rare peripheral lung tumor, characterized by the papillary growth of ciliated columnar, mucous, and basal cells. Our case was unusual because a growing cavitary lesion detected by computed tomography (CT) initially led us to suspect a colorectal cancer (CRC) metastasis.

## Case presentation

**Fig. 1:**
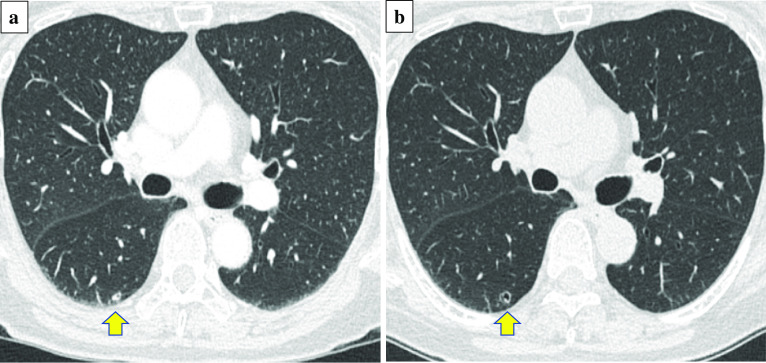
Serial CT findings of a ciliated muconodular papillary tumor (CMPT) at **a** colectomy performed 14 months prior and **b** thoracoscopic surgery. Yellow arrows indicate tumor growth from the nodule to a cavitary lesion suspicious of metastasis of colorectal cancer

A 70-year-old woman with a 45-year smoking history, who had undergone colectomy for CRC (pT3N0M0, p-stage II, well-differentiated tubular adenocarcinoma [tub1, pT3/SS, ly0, v0, pH0]). 2 years prior, presented with a CT nodule shadow in her right lower pulmonary lobe that had grown from 0.2 cm at her right hemicolectomy (Fig. 1a) to 0.3 cm at 12 months, and to 0.5 cm at 14 months postoperatively (Fig. 1b). The serum levels of tumor markers were within their normal ranges; CEA, 2.9 ng/mL; CA19-9, 6.8 U/mL; CA125, 4.0 U/mL. Because this growing cavitary lesion with a slightly irregular wall thickness suggested CRC metastasis, a thoracoscopic resection was performed.

**Fig. 2:**
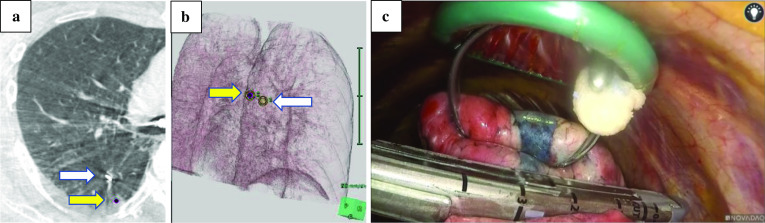
Intraoperative findings of thoracoscopic wedge resection using cone beam computed tomography (CT) in a hybrid surgical theater. **a** Intraoperative CT image. The yellow arrow indicates the tumor shadow position, and the white arrow denotes the tip of the injection catheter advanced through a working channel of a flexible bronchoscopy. **b** Three-dimensional positional relationship between the tumor (yellow arrow) and the marking spot on the pleural surface (white arrow), which was reconstructed in a surgical room. **c** Wedge resection was performed using a linear stapler

**Fig. 3:**
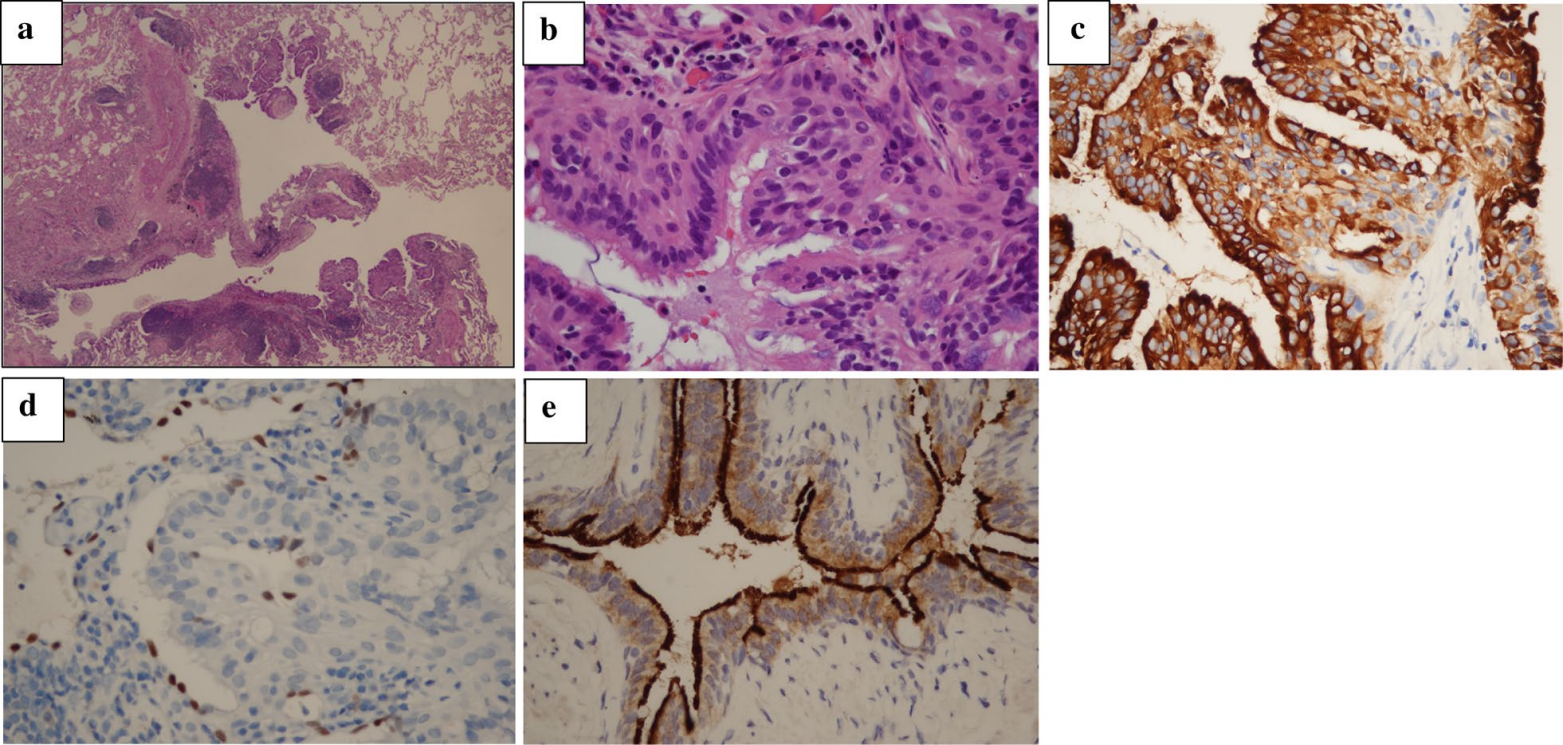
**a** Cyst cavity showing proliferation of papillary cells with cilia adjacent to the bronchi and mucous glands around the cystic wall (HE, ×20). **b** Mucous gland suggests alveolar structures were intact and composed of normal epithelial cells with no atypical cells (HE, ×200) in the resected tumor. **c** CK7 staining was strong and diffuse in most epithelial cells (×400). **d** Focal TTF-1 staining in occasional ciliated cells (×400). **e** Cilia cells were positive for BRAF staining (×400)

As the lesion was barely palpable, preoperative marking was required. Although the dye material was radiolucent, the tip of the catheter used for dye marking was radiopaque. After confirming the positional relationship between the tumor and the tip of the catheter by using cone-beam CT in the hybrid operating room, dye marking with indigo-carmine dye (0.3 mL) by bronchoscopy was performed at a point around 5 mm cranial to the tumor. Preoperative marking took 6 min with two CT inspections (Fig. 2a, b). Intraoperatively, the tumor had no gross pleural changes, but because it was slightly palpable at 5 mm caudal from the dyed site, we performed a wedge resection (Fig. 2c). Intraoperative frozen sections revealed fibrotic tissues with no apparent malignant cells, which did not match the CRC tissue. Permanent sections showed proliferating papillary cells with cilia adjacent to the bronchi and mucous glands around the cystic wall; the alveolar structure was intact and was composed of normal epithelial cells, with no atypical cells (Fig. 3a, b). Immunohistochemically, the lesion was positive for cytokeratin-7 (CK7), focal positive for thyroid transcription factor-1 (TTF-1), and negative for cytokeratin-20 (CK20) and caudal type homeobox-2 (CDX2), which ruled out CRC metastasis (Fig. 3c, d). We therefore diagnosed the tumor as CMPT. The patient has remained recurrence-free for 7 months.

## Discussion

Since CMPT was first reported in 2002 by Ishikawa et al., only 60 cases have been reported and it has not been categorized by the World Health Organization [1, 2] (Table 1). CMPT reportedly occurs more frequently in women (males:females, 1:1.2), and has no correlation with smoking history. Although it usually presents as a peripheral nodule with ground glass opacity (GGO) on CT (median size, 10 mm; range, 4–45 mm), only 13% of reported cases show central cavitation on CT, and a recent case series (n = 16) to assess thin-section CT features of CMPT showed no cases of cavity formation [3].

**Table 1:** Clinical features of reported CMPT cases

Author	Age	Sex	Location	CT finding	Size (mm)	Operative procedure	Intraoperative pathological diagnosis	Outcome (months)
Ishikawa(2002)	50	F	RUL	Nodule	15	L	n/a	120
Harada (2008)	62	M	LLL	Nodule	9	W	n/a	n/a
Sato (2010)	67	M	RUL	Nodule with GGO	5	W	Low-grade malignant tumor	10
	59	F	RLL	GGO with cavity	7	W	CMPT	18
Ishikawa (2013)	57	F	LLL	Nodule with cavity	11	L	Adenocarcinoma	6
Yuki (2013)	70	F	RLL	Nodule	8	W	n/a	n/a
Hata (2013)	76	F	LUL	Nodule	7	L	Malignancy	24
Chuang (2014)	68	M	RLL	Nodule	12	W	Adenocarcinoma	48
Kamata (2015)	61	M	RUL	Nodule	10	W	n/a	76
	60	F	LLL	Nodule	15	W	n/a	33
	78	M	RLL	Nodule	9	S	n/a	66
	63	M	RLL	Nodule	11	L	n/a	63
	75	M	LLL	Nodule	6	W	n/a	44
	62	F	LLL	Nodule with cavity	13	W	n/a	45
	57	M	RLL	Nodule	12	W	n/a	7
	56	M	RLL	Nodule	11	W	n/a	4
	66	M	LLL	Nodule	7	W	n/a	88
	61	F	RLL	Nodule	6	W	n/a	2
Chu (2015)	56	M	LUL	Nodule	11	S	Mucinous adenocarcinoma	5
Lau (2016)	19	F	RLL	Nodule with cavity	13	W	Mucinous neoplasm	n/a
Ishikawa (2016)	66	M	RUL	Nodule	13	L	Mucinous cystic neoplasm	58
	82	F	LLL	Nodule	10	W	No malignancy	55
	77	M	LLL	Mass with cavity	45	L	Adenocarcinoma suspected	48
	70	M	RLL	GGO	35	W	CMPT	19
	67	F	RLL	Nodule	5	W	No malignancy	28
Liu (2016)	60	M	RLL	Nodule	12	W	n/a	7
	83	F	RML	Nodule	4	L	No malignancy	n/a
	81	F	n/a	Nodule	4	W	No malignancy	n/a
	71	F	LUL	Nodule with GGO	12	W	Glandular papilloma	120
Kon (2016)	80	M	LLL	Nodule with cavity	7	W	n/a	29
	67	M	RLL	Nodule	10	W	n/a	25
	66	M	RLL	Nodule with cavity	13	L	n/a	14
	73	F	LUL	Nodule with cavity	9	W	n/a	5
	70	F	RLL	Nodule	8	W	n/a	48
Taguchi (2017)	84	F	RLL	Nodule	8	W	n/a	10
Segawa(2017)	42	M	LLL	Nodule with cavity	11	L	Mucinous adenocarcinoma	24
Jin (2017)	59	F	RLL	Nodule with cavity	8	L	Atypical glandular lesion	6
Udo (2017)	n/a (median 67)	F	n/a	n/a	n/a (median 11)	n/a (L3, S1)	n/a	n/a
	n/a	F	n/a	n/a	n/a	n/a	n/a	n/a
	n/a	F	n/a	n/a	n/a	n/a	n/a	n/a
	n/a	F	n/a	n/a	n/a	n/a	n/a	n/a
Kita (2018)	67	F	LLL	Nodule	7	W	No malignancy	24
Miyai (2018)	67	F	RML	Nodule with GGO	20	W	n/a	4
Shen (2019)	58	M	RLL	Nodule	11	L	Papillary carcinoma	n/a
	64	F	LLL	Nodule	8.5	W	Adenocarcinoma	n/a
Matsuoka (2019)	76	F	RLL	Nodule	10	W	Mucinous adenocarcinoma	24
Yao (2019)	67	F	LUL	Nodule	12	S	No malignancy	10
Cheung (2019)	61	M	RLL	Nodule with cavity	10	L	Mucinous adenocarcinoma	12
Shao (2019)	58	F	LLL	Nodule with GGO	8	W	n/a	n/a
	66	F	RLL	Nodule	6	W	n/a	n/a
Our case	70	F	RLL	Nodule with cavity	6	W	No malignancy	7

We did not add the 16 cases reported by Onishi et al. to the table at this time because the details of the clinical findings of the patients had not been described in the text

*M* male, *F*: female, *RUL* right upper lobe, *RML* right middle lobe, *RLL* right lower lobe, *LUL* left upper lobe, *LLL* left lower lobe, *GGO* ground glass opacity, *L* lobectomy, *S* segmental resection, *W* wedge resection, *CMPT* ciliated muconodular papillary tumor, *n/a* not applicable

Our case presenting with a growing cavity shadow was radiographically suspected to be CRC metastasis, because necrotic components, known as dirty necrosis, which suggest a colorectal origin, were considered to be drained through the airway [4]. However, the specimen was CK7 + /TTF1 + /CDX2 − /CK20 − , which indicated that it was not a metastasis. CMPT typically shows distinct papillary growth of a mixture of ciliated columnar, mucous, and basal cells, often with central mucin accumulation, focal fibrosis, and a disrupted alveolar framework, which correlates with cavitation on CT. Because differential diagnosis of CMPT includes adenocarcinoma with cilia formation, mucinous adenocarcinoma, mucoepidermoid carcinoma, peribronchiolar metaplasia, and glandular papilloma, intraoperative diagnosis of these lesions from a small specimen can be challenging. To our knowledge, only two CMPT cases have been diagnosed intraoperatively, both of which by facilities that had previously diagnosed CMPT [5]. Histopathologically, our CMPT was diagnosed as a benign lesion; however, some reports suggest it to be a precursor of adenocarcinoma because they had confirmed BRAF, EGFR, and ALK mutations, which occur early in lung adenocarcinogenesis. In our case, BRAF immunostaining was positive for only cilia cell, and the tumor itself was not stained (Fig. 3e); however, other studies reported that epithelial cells and cytoplasm had been stained [6, 7]. Because CMPT is rare and lacks accumulated studies, whether these molecular findings support CMPT being an adenocarcinoma precursor remains unclear.

Regarding optimal resection, in a thoracoscopic setting without any preoperative marking, accurate localization of the 6-mm tumor located apart from the pleura seemed to be difficult [8, 9]. Generally, preoperative marking methods for small lesions include CT-guided marking and bronchoscopic marking. However, CT-guided marking with hook wires can cause pneumothorax, bleeding, and potentially fatal air embolism in about 1.3% of cases [10]. On the other hand, bronchoscopic marking has a lower risk of complications compared to the former method, but if the lesion is too faint to be identified using fluoroscopy or when it located deep to the pleura, the marking procedure itself tends to be difficult because the positional relationship between the marking position and the tumor cannot be grasped. In this case, because the lesion seemed to be difficult to detect by fluoroscopy and could not be palpated because of the small size and a morphology of the cavity, we planned a more reliable and less invasive preoperative marking following resection using a cone-beam CT in a hybrid operating room. A quarter of reported CMPT cases were treated with lobectomies despite the small lesion size. Despite the potential malignancy of CMPT, no recurrence or metastasis has been reported for up to 10 years by wedge resection, and thus additional resection was not planned after the final diagnosis of CMPT.

## Conclusions

CMPT can present as GGO, nodules, or (rarely) cavitary formation with irregular wall thickness mimicking a metastasis. Its pathology is not clearly defined, and it may have benign or malignant properties, depending on the molecular alterations. Although no recurrence or metastasis has been reported, CMPT should be resected with sufficient margins.

## Data Availability

Data sharing is not applicable to this article as no datasets were generated or analyzed during the current study.

## Abbreviations

CMPT: Ciliated muconodular papillary tumor
CT: Computed tomography
CRC: Colorectal cancer
CK7: Cytokeratin-7
CDX2: Caudal type homeobox-2
GGO: Ground glass opacity
EGFR: Epidermal growth factor receptor
ALK: Anaplastic lymphoma kinase
